# Epoxide based inhibitors of the hepatitis C virus non-structural 2 autoprotease

**DOI:** 10.1016/j.antiviral.2015.02.005

**Published:** 2015-05

**Authors:** Joseph Shaw, Colin W.G. Fishwick, Mark Harris

**Affiliations:** a School of Molecular and Cellular Biology, Faculty of Biological Sciences, University of Leeds, Leeds LS2 9JT, United Kingdom; b School of Chemistry, University of Leeds, Leeds LS2 9JT, United Kingdom; c Astbury Centre for Structural Molecular Biology, University of Leeds, Leeds, LS2 9JT, United Kingdom

**Keywords:** HCV, hepatitis C virus, NS, non-structural, SGR, subgenomic replicon, TPCK, tosyl phenylalanine chloromethyl ketone, GdnHCl, guanidine hydrochloride, CHAPS, 3-[(3-cholamidopropyl)dimethylammonio]-1-propanesulphonate, DTT, dithiothreitol, JFH1, Japanese Fulminant Hepatitis 1, EC_50_, 50% effective concentration, LarI, luciferase assay reagent 1, IMAC, immobilised metal ion affinity chromatography, ffLuc–NPT, firefly luciferase–neomycin phosphotransferase, Hepatitis C virus, NS2, Inhibitor, Autoprotease, Epoxide

## Abstract

Hepatitis C virus (HCV) non-structural 2 (NS2) encodes an essential protease activity responsible for processing at the NS2–NS3 junction which represents an attractive antiviral target. Attempts to inhibit the NS2 autoprotease with mechanism-based protease inhibitors and substrate peptides have had limited success. We report a series of epoxide-containing small molecules capable of blocking NS2–NS3 proteolysis *in vitro* and demonstrate the potential for selectivity towards the NS2 autoprotease. A compound within this series was able to perturb HCV genome replication in a subgenomic replicon system only when polyprotein processing was dependent on NS2 autoprotease activity, in addition it inhibited replication of full length HCV. These findings suggest blocking HCV polyprotein processing through inhibition of the NS2 autoprotease represents a viable route to exert an antiviral effect.

## Highlights

• Epoxide-containing small molecules inhibit hepatitis C virus NS2 autoprotease *in vitro*.
• These molecules also show specific inhibition of NS2–NS3 autocleavage dependent HCV genome replication in a cell based assay.
• NS2 protease is a valid target for further drug development.

## 1 Introduction

Hepatitis C virus (HCV) is a global health burden infecting 3% of the world’s population. As a positive sense RNA virus within the *Flaviviridae* family HCV relies on proteolytic processing of a single polyprotein to generate mature proteins. The structural proteins Core and E1–E2, as well as p7, are processed by host proteases, while the non-structural (NS) proteins responsible for genome replication undergo maturation by virally encoded proteases. Autoproteolysis occurs at the NS2–NS3 boundary via a cysteine protease activity encoded principally within NS2 but enhanced by the presence of the NS3 N-terminus (Schregel et al., 2009). NS3 with its cofactor NS4A (NS3-4A) mediates subsequent downstream cleavages to generate NS4B, NS5A and NS5B (Scheel and Rice, 2013).

Inhibitors of the NS3-4A protease that disrupt polyprotein processing are now approved for the treatment of HCV infection. However, NS2 protease activity remains an unexplored target. NS2 plays no direct roles in genome replication, as demonstrated by the ability of a subgenomic replicon (SGR) to replicate in the absence of NS2 (Lohmann et al., 1999). However, the unprocessed NS2–NS3 precursor has reduced NS3 protease activity, potentially by reducing NS3 proteolysis kinetics or through reduced stability of NS3 (Welbourn et al., 2005). Hence where NS3 is derived from a NS2–NS3 precursor, as in the context of infectious virus, the activity of the NS2 autoprotease is essential (Jones et al., 2007; Kolykhalov et al., 2000). Mutational analysis and structural studies of the post-cleavage NS2 protease domain propose that NS2 acts as a cysteine protease, though the catalytic triad appears to adopt the geometry of a serine protease (Lorenz et al., 2006). Due to the essential nature of the NS2 autoprotease it has been proposed as an attractive target for antivirals that to date has not been explored (Rice, 2011).

A common route to develop a protease inhibitor is to incorporate an electrophilic ‘warhead’ so as to produce a mechanism-based inhibitor (Powers et al., 2002). Such reactive warheads form an irreversible covalent interaction with the active site residues, but often lack selectivity. In contrast, an epoxide warhead forms a covalent interaction with the nucleophilic catalytic residue only when the epoxide is held non-covalently in the optimal orientation. As such the rate limiting step in protease inhibition by epoxides is the formation of a non-covalent binding pose so as to optimally orientate the epoxide for nucleophilic attack (Bihovsky et al., 1993). This transient interaction is usually mediated by a conjugated substrate peptide derivative and can be tailored to the system, allowing epoxide-based protease inhibitors a greater degree of selectivity (Powers et al., 2002). However, unlike the HCV NS3-4A protease, which is inhibited by peptides corresponding to the N-terminus of the cleavage site (Llinas-Brunet et al., 1998), the NS2 autoprotease shows little or no sensitivity to substrate or proteolysis product peptides *in vitro*, nor to substrate peptides conjugated to mechanism-based protease inhibitors (Pallaoro et al., 2001; Thibeault et al., 2001). In addition, the cysteine protease inhibitor E64, an epoxysuccinyl peptide, has proven inactive against the NS2 autoprotease (Pieroni et al., 1997; Thibeault et al., 2001). We therefore sought to explore the activity of epoxide warheads conjugated to non-peptide derivatives, in particular a range of reported *cis*-bisamido epoxides conjugated to aromatic ‘backbones’ which show activity against the cysteine containing active site of factor XIIIa (Avery et al., manuscript submitted). Derivatives of this scaffold showed activity against the NS2 autoprotease *in vitro*. Importantly, the epoxide alone was insufficient for activity and variations to the aromatic backbone altered inhibitory activity, indicating the requirement for a transient interaction and suggesting epoxide based inhibitors can be tailored to the NS2 active site. Epoxide based inhibitors of the NS2 autoprotease exerted an effect in cell based assays in which HCV genome replication was NS2-dependent. To the best of our knowledge these findings represent the first example of direct pharmacological inhibition of the NS2 autoprotease to block HCV genome replication and exert an antiviral effect.

## 2 Materials and methods

### 2.1 Compounds

Synthesis and analysis of compounds **1**–**10** has been reported elsewhere (Avery et al., manuscript submitted). Tosyl phenylalanine chloromethyl ketone (TPCK) was from VWR International. E64 and Telaprevir were from Sigma Aldrich and MedChem Express respectively. All compounds were confirmed by mass spectrometry analysis using a VG Autospec mass spectrometer with electron spray ionisation (ES) at 70 eV.

### 2.2 NS2–3 auto-processing assay

Expression of NS2–NS3-FLAG in pET23a has been previously described (Foster et al., 2010; Tedbury and Harris, 2007) (Supplementary material). NS2–3 at 20 μM in 6 M GdnHCl was diluted 1:100 into Refolding buffer (30% glycerol, 0.5% CHAPS, 250 mM NaCl, 3 mM cysteine, 50 μM ZnCl_2_, 10 mM DTT, 50 mM HEPES–NaOH, pH 7.0) and incubated 16 h RT. Compounds (133 × final concentration in DMSO) were diluted into Refolding buffer before the addition of NS2–3 to yield required final concentrations. Reactions were halted by the addition of ¼ volume 4 × Laemmli buffer (200 mM Tris–HCl pH 6.8, 40% glycerol, 8% SDS, 0.08% bromophenol blue, 20 mM DTT) and analysed by 15% SDS–PAGE and western blot using M2 anti-FLAG monoclonal antibody (Sigma Aldrich) followed by IRDye 680RD Donkey anti-Mouse secondary (LI-COR Biosciences). Imaging and analysis was performed using Odyssey imager (LI-COR Biosciences). Intensity of the 20 kDa proteolysis product (NS3-FLAG) was quantified to calculate 50% effective concentration (EC_50_) using Prism 6 (GraphPad).

### 2.3 SGR compound treatments

Stable cell lines harbouring SGR (Supplementary material) were maintained in Dulbecco’s modified Eagle’s medium (DMEM; Sigma) supplemented with 10% foetal bovine serum (FBS), 100 IU penicillin ml^−1^, 100 μg streptomycin ml^−1^, 1% non-essential amino acids and 300 μg/ml G418 (Sigma) in a humidified incubator at 37 °C in 5% CO_2_. Cells were seeded at a density of 2 × 10^4^ cells/well in a 96 well plate. After 16 h media was removed and replaced with media supplemented with the indicated concentrations of compounds with standard DMSO at 0.25%. Plates were incubated in the absence of G418 for a further 48 h.

### 2.4 Firefly luciferase endpoint assay

Luciferase activity was quantified by addition of 30 μl Passive Lysis Buffer (PLB: Promega) and addition of 40 μl LarI reagent (Promega) using a BMG Labtech plate reader with light emission recorded over 6 s. Data was normalised to DMSO control. Statistical significance was determined using the Student’s *t* test.

### 2.5 Cell viability endpoint assay

Cellular metabolism was quantified by 2 h incubation in 1 mM Thiazolyl Blue Tetrazolium Bromide (Sigma Aldrich) before crystals were suspended in 100 μl DMSO and absorbance at 570 nm measured using an infinite F50 platereader (Tecan). Data was normalised to DMSO control. CC_50_ was calculated using Prism 6 (GraphPad).

### 2.6 HCVcc compound treatments

Transcripts (5 μg) of a Jc1 derivative expressing Nanoluciferase (JC1-NLuc) (Amako et al., 2015) were electroporated into Huh7 cells (see Supplementary). Compound was added as in Section 2.3. Cell viability was performed as in Section 2.5 following 4% paraformaldehyde fixation of cells. NanoLuc was measured using a BMG Labtech plate reader following addition of 50 μl PLB and addition of equal volume of NanoGlo Luciferase Assay Substrate (Promega).

## 3 Results

### 3.1 The NS2 autoprotease is inhibited by halomethyl ketones but not the epoxide-based inhibitor E64

To assess the ability of a small molecule to inhibit NS2 protease activity, an *in vitro* auto-processing assay was employed. A NS2–NS3 precursor protein (NS2–3) comprising the catalytic C-terminal domain of NS2 and the N-terminal protease domain of NS3 (JFH1 polyprotein residues 906–1209, J4 residues 904–1206) flanked by an N-terminal His tag and C-terminal FLAG tag was bacterially expressed and purified from inclusion bodies under denaturing conditions by immobilised metal ion affinity chromatography (IMAC). Upon dilution into Refolding buffer, NS2–3 forms significant secondary structure (Foster et al., 2010) allowing the autoprotease to become active. This can be monitored by western blot analysis of NS2–3 refolding reactions with an anti-FLAG antibody to reveal 35 kDa precursor NS2–3-FLAG and 20 kDa NS3-FLAG, one of the proteolysis products. Quantification of the proteolysis product was used as a relative measure of NS2 autoprotease activity.

**Fig. 1:**
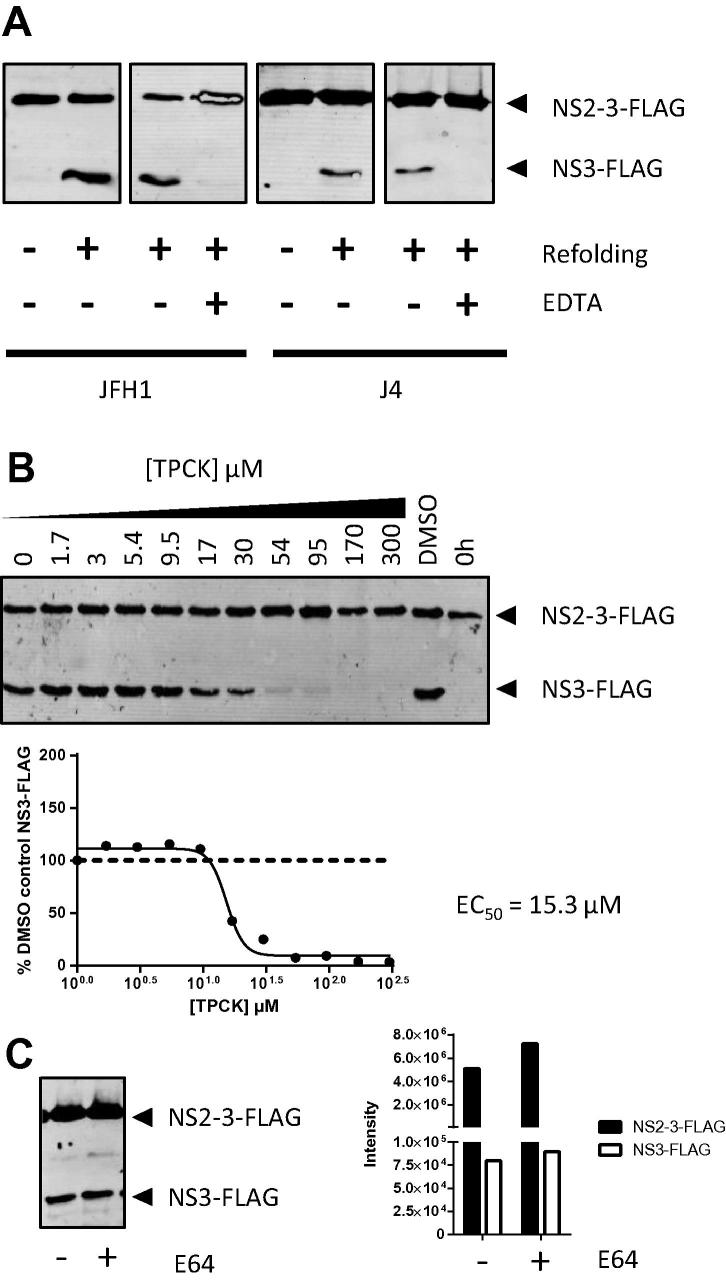
*In vitro* NS2–3 refolding to assay NS2 autoprotease activity. (A) Purified NS2–3 of the genotype 2a isolate JFH1 (left panel) and genotype 1b isolate J4 (right panel) produce detectable NS3-FLAG proteolysis product upon refolding. Addition of EDTA (10 mM) blocks autoproteolysis-induced NS3-FLAG production. (B) JFH1 NS2–3 autoproteolysis was treated with indicated concentrations of tosyl phenylalanine chloromethyl ketone (TPCK) and compared to a reaction halted before refolding (0 h) and DMSO (0.75%) control. EC_50_ was calculated from quantified NS3-FLAG normalised to DMSO control (plotted as 10^0^ μM). (C) JFH1 NS2–3 autoproteolysis produced comparable NS3-FLAG proteolysis product in the absence (−) or presence (+) of E64 (300 μM).

Purified NS2–3 of the genotype 2a isolate JFH1 and the genotype 1b isolate J4 were both capable of auto-processing under these conditions. NS2 autoprotease activity was inhibited by EDTA (Fig. 1A), consistent with a structural requirement for zinc. As previously reported (Pallaoro et al., 2001; Pieroni et al., 1997; Thibeault et al., 2001), autoprotease activity was inhibited by the halomethyl ketone tosyl phenylalanine chloromethyl ketone (TPCK) (Fig. 1B) with an EC_50_ of 15.3 μM. NS2 was not inhibited by E64 (Fig. 1C), confirming previous observations (Pieroni et al., 1997; Thibeault et al., 2001).

### 3.2 Aromatic bis-amido based epoxides as inhibitors of the NS2 autoprotease

To test the ability of non-peptide derived epoxides to inhibit the NS2 autoprotease, a range of previously reported compounds containing an epoxide conjugated to a functionalised benzene ring (Avery et al., manuscript submitted) were tested in the *in vitro* auto-processing assay. Structures for these compounds are shown in Table 1.

**Fig. 2:**
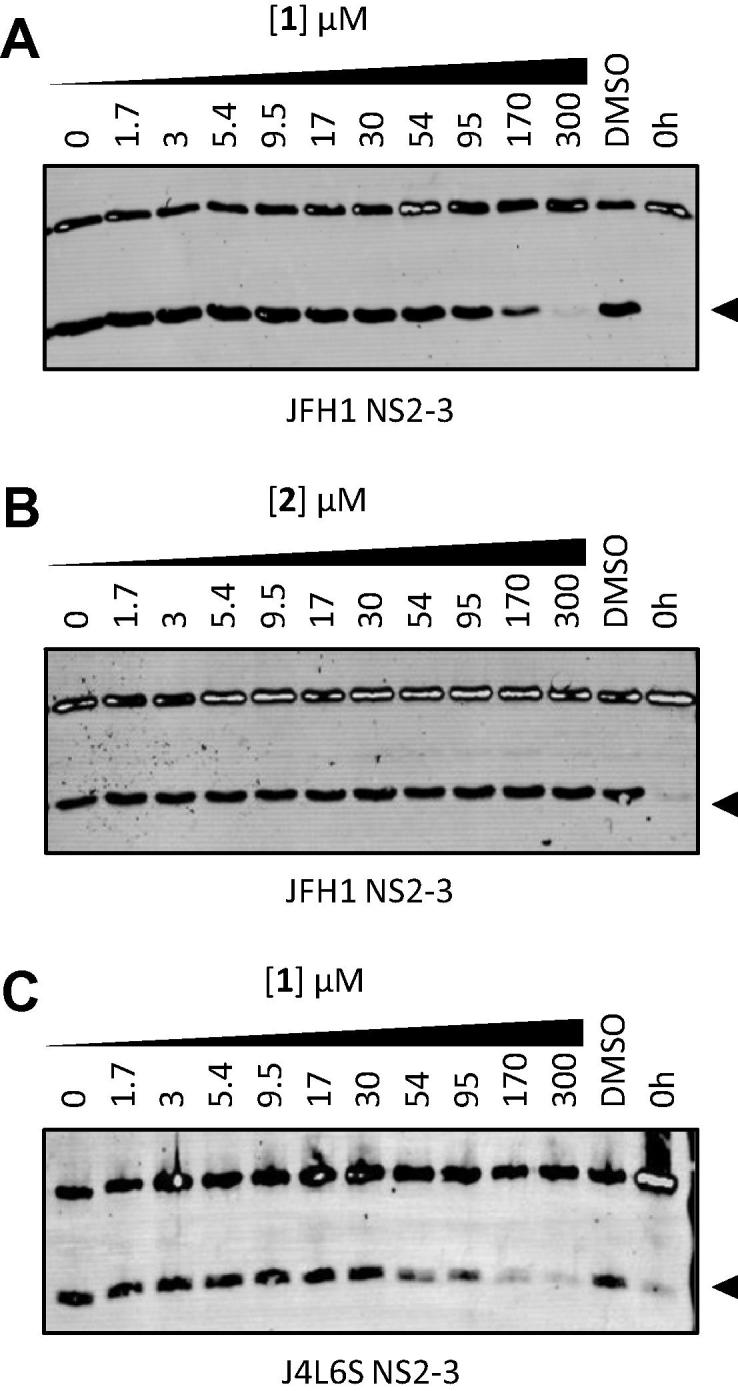
Epoxide based inhibitors of the NS2 autoprotease. (A) JFH1 NS2–3 refolding reactions were treated with indicated concentrations of **1** alongside controls and NS3-FLAG proteolysis product (arrowhead) was quantified by western blot. EC_50_ = 92 μM. (B) Identical treatment of JFH1 NS2–3 refolding reactions with **2**. EC_50_ = >300 μM. (C) J4 NS2–3 refolding reactions treated as in A. EC_50_ = 79 μM. Chemical structures are shown in Table 1.

Compound **1** was identified as capable of blocking *in vitro* NS2–NS3 processing in a dose responsive manner for both JFH1 and J4 isolates, with J4 showing enhanced sensitivity (Fig. 2). Interestingly, a derivative lacking only the benzene 3-chloro group (**2**) appeared inactive up to 300 μM (Fig. 2B). This finding indicated that elements other than the epoxide alone contributed to the inhibitory effect observed.

To further explore the contributions of the benzene substituents to activity, a range of compounds containing the same epoxide as **1** with alternate R_1_ groups (Table 1) were tested against the JFH1 NS2–3 auto-processing assay. These molecules are all active to varying degrees against factor XIIIa (Avery et al., manuscript submitted), and are derived from the anti-factor XIIIa activity of the epoxide-based natural product cerulenin, which contains an extended hydrocarbon chain. Cerulenin showed no activity against the NS2 autoprotease (Table 1). Activities of these compounds (**3**–**9**) are shown in Table 1.

A number of chemical modifications reduced activity of **1** to below the level of detection – these included removal of the 3-chloro substituent from the benzene (**2**), introduction of a 4-bromo substituent without (**3**) or with the 3-chloro group (**4**) and presence of 3,4-dimethyl groups (**5**). Compounds **7** and **8**, with a 4-amino-phenyloxy or 4-nitro-phenyloxy substituent respectively, showed some activity but to a lesser extent to that shown by **1**, though a 3-phenyloxy substituent did enhance activity (**6**). The most potent inhibitor was a 4-benzyloxy, 3-chloro substituted derivative (**9**) yielding an EC_50_ of 55 μM. The varying activity of **1**–**9** demonstrates the ability of the compound backbone to modulate activity. In agreement with this, the epoxide alone, where the functionalised amido-benzene backbone is replaced by a primary alcohol (**10**), was inactive (Table 1, Supplementary Fig. 1).

### 3.3 Activity of epoxide based inhibitors in a cell based assay

In other systems, the requirement of epoxide-based inhibitors to form a transient interaction with the protease before becoming covalently linked allows them greater selectivity and makes them more amenable to *in vivo* applications (Powers et al., 2002). To explore the potential for bis-amido based epoxide warheads to exert an inhibitory effect within a HCV model system, compounds **1**–**10** and cerulenin were tested for cytotoxic effects by calculating CC_50_ from 72 h incubation in a hepatoma cell line capable of supporting HCV replication (Supplementary Fig. 2). Of the compounds showing significant activity *in vitro* only **1** and **7** exerted no significant cytotoxicity up to 100 μM (Table 1). The activity of **1** in cell based assays was further explored due to the availability of **2** which, as an inactive derivative without significant cytotoxic effects, allows a comparative tool.

**Fig. 3:**
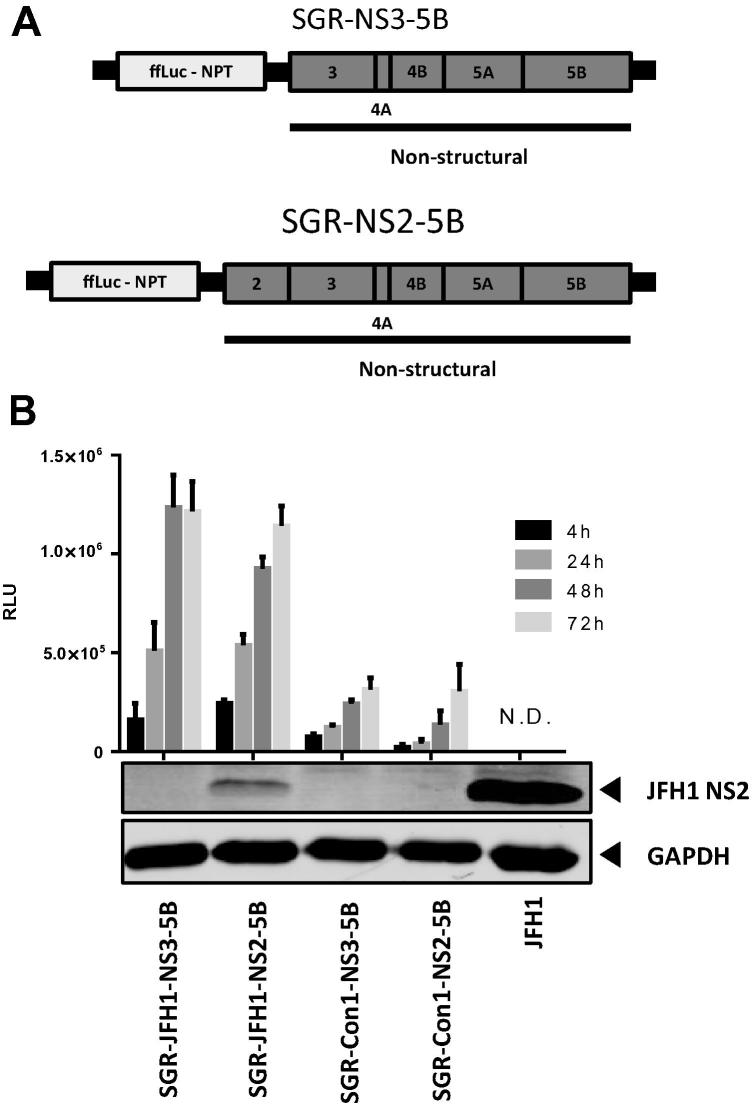
Comparative analysis of sub-genomic replicon (SGR) stable cell lines. (A) Schematic of firefly luciferase–neomycin phosphotransferase (ffLuc–NPT) reporter SGRs comprising NS3-5B or NS2-5B. (B) Luciferase activity (RLU – relative luciferase units) from stable SGR harbouring cell lines analysed after indicated timepoints post seeding (N.D. – not determined). Data represents the average of three technical repeats ± SD. Lysates were analysed by western blot and probed with anti-JFH1 NS2 antibody and anti-GAPDH antibody alongside a control lysate from JFH1 infected cells.

In order to test for specific activity against NS2-dependent HCV replication, stable cell lines were selected that harbour a HCV SGR comprising either NS2-5B or NS3-5B of the JFH1 or Con1 isolates (Fig. 3A). These replicons contain a fused firefly luciferase neomycin phosphotransferase-reporter enabling both stable selection and luciferase production as a measure of HCV replication (Wyles et al., 2009). All replicon cell lines displayed reported sensitivity to cyclosporine A (data not shown). Replication kinetics as measured by luciferase activity were comparable in the absence or presence of NS2 for both genotypes (Fig. 3B). Western blot confirmed the presence of mature NS2 (JFH1 strain only due to genotype-specific antibody reactivity) while sequence analysis of SGR RNA extracted from the stable cell lines did not reveal any additional culture adaptive mutations.

**Fig. 4:**
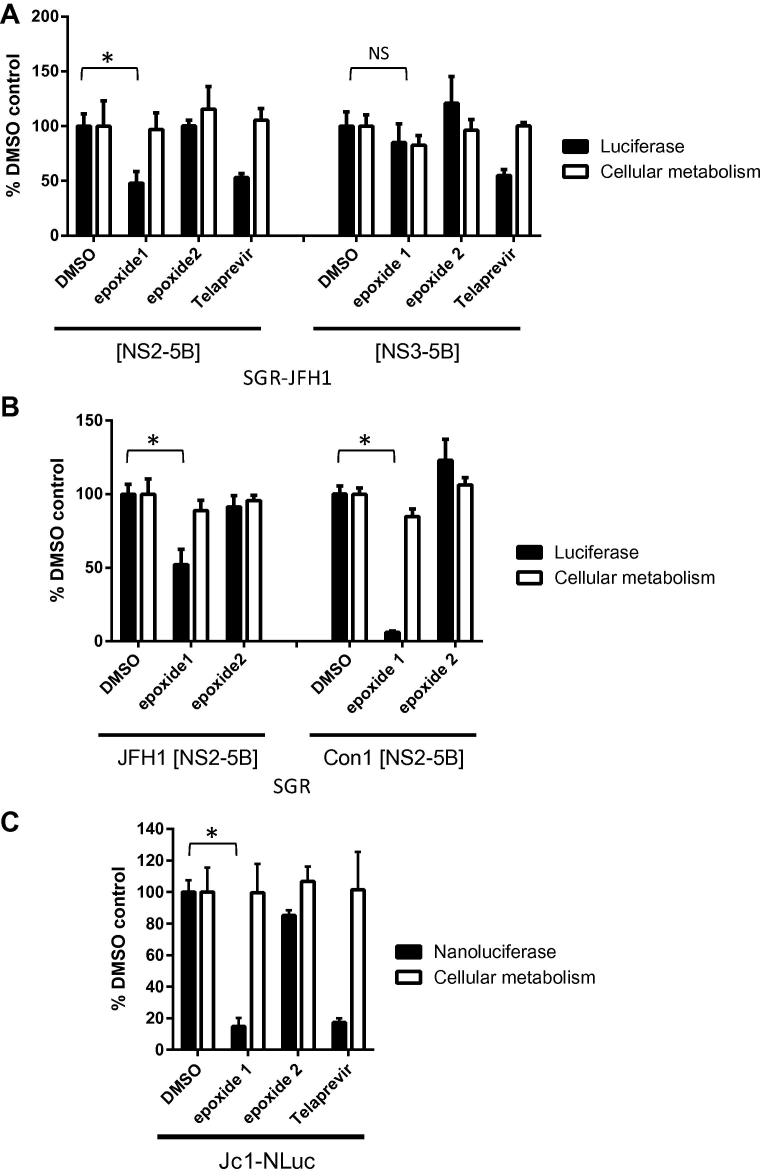
Cell based activity of epoxide based inhibitors of the NS2 autoprotease. (A) SGR-JFH1 cell lines containing ([NS2-5B]) or lacking ([NS3-5B]) NS2 were treated in parallel with **1** (90 μM) or **2** (90 μM). Luciferase activity and cellular metabolism were normalised to DMSO control. Values and error bars represent the mean and standard deviation of two independent experiments carried out in triplicate. Both cell lines were treated in parallel with Telaprevir (150 nM) (one technical repeat carried out in triplicate). (B) SGR-NS2-5B derived from the genotype 2a isolate JFH1 or the genotype 1b isolate Con1 were treated in parallel with **1** (90 μM). Luciferase activity and cellular metabolism were normalised to DMSO control. Values and error bars represent the mean and standard deviation of three independent experiments carried out in triplicate. Both cell lines were treated in parallel with **2** (90 μM) (one technical repeat carried out in triplicate). (C) Jc1-NLuc was electroporated into Huh7 cells and treated at 4 h.p.e with epoxide 1 (90 μM) or epoxide 2 (90 μM). At 48 h.p.e NLuc activity and cellular metabolism were quantified. Values are normalised to DMSO control and represent the mean and standard deviation of two experimental repeats performed in triplicate. Jc1-NLuc was also treated with Telaprevir (150 nM) (one technical repeat carried out in triplicate). Statistical significance was determined by the Student’s *t* test (^∗^*p* < 0.0001).

To assess activity against HCV genome replication, SGR-JFH1-NS2-5B was treated with **1** (at *in vitro* EC_50_90 μM) and the same concentration of the inactive derivative **2**. Quantification of luciferase activity revealed reduction to 48.1 ± 10.7% of vector control upon treatment with **1** (Fig. 4A), whereas **2** had no effect. Cell viability was unaffected by **1**, indicative that the decreased luciferase activity represented a specific inhibitory effect against genome replication. The same treatment was performed in parallel against SGR-JFH1-NS3-5B, which lacks the NS2 coding sequence and can therefore support HCV replication independent of NS2 autoprotease activity. Treatment of this replicon cell line with **1** (90 μM) produced a slight decrease in luciferase activity which correlated with a decrease in cellular metabolism and was not statistically significant, indicating that **1** exerts no inhibitory activity against NS2-independent HCV replication (Fig. 4A). To confirm there are no significant differences between the response to inhibitor treatment across the two cell lines, both were treated with an intermediate concentration of the NS3-4A protease inhibitor Telaprevir (Kwong et al., 2011). For both SGR-JFH1-NS2-5B and SGR-JFH1-NS3-5B, addition of Telaprevir (150 nM) produced a comparable reduction in luciferase activity with no effect on cellular metabolism (Fig. 4A).

*In vitro* activity of **1** against the NS2 autoprotease had indicated an increased potency against the genotype 1b J4 isolate (Fig. 2). To explore whether this difference in genotype sensitivity was observed in the context of HCV genome replication, **1** was tested in parallel against SGR-JFH1-NS2-5B and SGR-Con1-NS2-5B. While treatment with **1** (90 μM) produced a 47.8 ± 10.3% effect against JFH1 SGR, the same treatment of a Con1 SGR reduced luciferase activity by 94 ± 1%, with a similar toxicity profile (Fig. 4B). As with SGR-JFH1-NS2-5B, SGR-Con1-NS2-5B showed no sensitivity to treatment with **2**.

Finally, epoxides **1** and **2** were tested against a chimeric infectious clone derived from genotype 2a isolates; Jc1 and containing a Nanoluc reporter gene (Jc1-NLuc). Following electroporation of Jc1-NLuc RNA into Huh7 cells treatment with **1** reduced reporter activity to 14.8 ± 5.4% of control (Fig. 4C). Hence in the context of a transient assay with infectious HCV, **1** exhibited enhanced antiviral activity compared to a stable SGR system. This effect most likely reflects the differences between transient and stable systems for monitoring HCV replication, as it was also observed upon treatment with Telaprevir (Fig. 4).

## 4 Discussion

The NS2 autoprotease is essential in the HCV lifecycle and with no homology to any human proteases represents an attractive drug target. Recent evidence suggests processing of the NS2–NS3 junction may represent a rate limiting step in the onset of HCV genome replication (Madan et al., 2014), hence the virus lifecycle may prove particularly susceptible to inhibition of the NS2 autoprotease. Despite concerted efforts, identification of NS2 inhibitors has been hampered by weak activities of classical protease inhibitors and substrate peptides. Mechanism-based protease inhibitors conjugated to peptide derivatives, such as E64, or to NS2 substrate peptides have shown no inhibitory activity, suggesting the modification of peptide substrates may not be a viable route for the development of an NS2 autoprotease inhibitor. Here we report the use of bis-amido aromatic scaffolds conjugated to an epoxide warhead as inhibitors of the NS2 autoprotease. Treatment of NS2–3 auto-processing reactions with epoxides conjugated to small, non-peptide backbones reduced production of the NS3-FLAG proteolysis product in a dose responsive manner. Importantly, these inhibitory effects did not appear to be imparted solely by the epoxide, as modifications to the ‘backbone’ of epoxy-bis-amido derivatives altered activity. Such scaffolds might be modified to introduce selectivity to the NS2 autoprotease active site. While a degree of structure-relationship analysis has already been performed *in vitro* for this series, future work should attempt to increase the potency of inhibition before consideration as a clinical candidate.

Epoxide-derived mechanism-based inhibitors have been reported to show selectivity to cysteine proteases over serine or aspartyl proteases (Powers et al., 2002) and to specific target proteases within this class (Schiefer et al., 2013). In agreement, cerulenin and other epoxysuccinyl peptides display little reactivity to free thiols in the form of glutathione (Powers et al., 2002) (Avery et al., manuscript submitted). The observation of selectivity with these molecules suggest that epoxide warheads could be targeted to the NS2 autoprotease to increase both activity and selectivity through the use of a backbone scaffold not derived from peptide substrates. The inactivity of peptide conjugated mechanism-based inhibitors could be attributed to size, in line with the inactivity of cerulenin.

Observations made in the *in vitro* NS2–3 auto-processing assay were also made in the context of HCV genome replication. A concentration of **1** active against the NS2 autoprotease *in vitro* exhibited an inhibitory effect specific to NS2-dependent HCV genome replication whereas **2**, inactive *in vitro*, also showed no activity in the same SGR cell lines. Furthermore, a genotype difference in sensitivity to **1** was observed both *in vitro* and in the context of NS2-dependent HCV replication, where enhanced sensitivity was observed against genotype 1b compared to 2a. Compound **1** therefore appears more potent against genotype 1b than genotype 2a both *in vitro* and in HCV model systems and it would be of interest to extend this analysis to other genotypes. Encouragingly **1** showed increased activity against full length infectious HCV compared to that observed against stable SGR harbouring cells. This suggests that, as expected, preexisting replication complexes present in stable SGR-harbouring cells are resistant to NS2 inhibitors. Although the compounds described here are unlikely to be taken forward to the clinic, the current data represent the first reported example of direct pharmacological inhibition of the NS2 autoprotease to block HCV genome replication and exert an antiviral effect and thus provide proof of principle that the NS2 autoprotease is a valid target for future development.

## Figures and Tables

**Table 1:** Activity of a range of epoxide based inhibitors of NS2–3 autoproteolysis. EC_50_ calculated from 10-point dose response curve against JFH1 NS2–3 refolding reaction (Supplementary Fig. 1). CC_50_ calculated from 11-point dose response curve against Huh7 cells (Supplementary Fig. 2).


Compound #	R_1_	NS2–3 refolding EC_50_ (μM)	Huh7 CC_50_ (μM)
**1**		93	>100
**2**		>300	>100
**3**		>200	17
**4**		>200	2
**5**		>300	>100
**6**		70	42
**7**		96	>100
**8**		119	62
**9**		55	12.6
**10**		>300	>100
Cerulenin		>300	31.9

## Appendix A Supplementary data

Supplementary Fig. 1 Activity against *in vitro* autoprotease activity. JFH1 NS2–3 refolding reactions were treated with a range of concentrations of **1**–**10** or cerulenin and NS3-FLAG proteolysis product (arrowhead) quantified by western blot. Determined EC_50_ values are shown in Table 1.

Supplementary Fig. 2 Cytotoxicity analysis of epoxide-based inhibitors of the NS2 autoprotease. Huh7 cells treated with indicated concentrations of **1**–**10**, cerulenin or TPCK for 72 h (*n* = 2, average ± SD). Determined CC_50_ values for **1**–**10** and cerulenin are shown in Table 1. TPCK CC_50_ = 3.3 μM.

## References

[b9000] Amako Y., Munakata T., Kohara M., Siddiqui A., Peers C., Harris M. (2015). Hepatitis C virus attenuates mitochondrial lipid β-oxidation by down-regulating mitochondrial trifunctional protein expression. J Virol..

[b0005] Bihovsky R., Powers J.C., Kam C.M., Walton R., Loewi R.C. (1993). Further evidence for the importance of free carboxylate in epoxysuccinate inhibitors of thiol proteases. J. Enzyme Inhib..

[b0010] Foster T.L., Tedbury P.R., Pearson A.R., Harris M. (2010). A comparative analysis of the fluorescence properties of the wild-type and active site mutants of the hepatitis C virus autoprotease NS2–3. Biochim. Biophys. Acta.

[b0015] Jones C.T., Murray C.L., Eastman D.K., Tassello J., Rice C.M. (2007). Hepatitis C virus p7 and NS2 proteins are essential for production of infectious virus. J. Virol..

[b0020] Kolykhalov A.A., Mihalik K., Feinstone S.M., Rice C.M. (2000). Hepatitis C virus-encoded enzymatic activities and conserved RNA elements in the 3′ nontranslated region are essential for virus replication in vivo. J. Virol..

[b0025] Kwong A.D., Kauffman R.S., Hurter P., Mueller P. (2011). Discovery and development of telaprevir: an NS3–4A protease inhibitor for treating genotype 1 chronic hepatitis C virus. Nat. Biotechnol..

[b0030] Llinas-Brunet M., Bailey M., Fazal G., Goulet S., Halmos T., Laplante S., Maurice R., Poirier M., Poupart M.A., Thibeault D., Wernic D., Lamarre D. (1998). Peptide-based inhibitors of the hepatitis C virus serine protease. Bioorg. Med. Chem. Lett..

[b0035] Lohmann V., Korner F., Koch J., Herian U., Theilmann L., Bartenschlager R. (1999). Replication of subgenomic hepatitis C virus RNAs in a hepatoma cell line. Science.

[b0040] Lorenz I.C., Marcotrigiano J., Dentzer T.G., Rice C.M. (2006). Structure of the catalytic domain of the hepatitis C virus NS2–3 protease. Nature.

[b9005] Madan V., Paul D., Lohmann V., Bartenschlager R. (2014). Inhibition of HCV replication by cyclophilin antagonists is linked to replication fitness and occurs by inhibition of membranous web formation. Gastroenterology.

[b0045] Pallaoro M., Lahm A., Biasiol G., Brunetti M., Nardella C., Orsatti L., Bonelli F., Orru S., Narjes F., Steinkuhler C. (2001). Characterization of the hepatitis C virus NS2/3 processing reaction by using a purified precursor protein. J. Virol..

[b0050] Pieroni L., Santolini E., Fipaldini C., Pacini L., Migliaccio G., la Monica N. (1997). *In vitro* study of the NS2–3 protease of hepatitis C virus. J. Virol..

[b0055] Powers J.C., Asgian J.L., Ekici O.D., James K.E. (2002). Irreversible inhibitors of serine, cysteine, and threonine proteases. Chem. Rev..

[b0060] Rice C.M. (2011). New insights into HCV replication: potential antiviral targets. Top Antivir. Med..

[b0065] Scheel T.K., Rice C.M. (2013). Understanding the hepatitis C virus life cycle paves the way for highly effective therapies. Nat. Med..

[b0070] Schiefer I.T., Tapadar S., Litosh V., Siklos M., Scism R., Wijewickrama G.T., Chandrasena E.P., Sinha V., Tavassoli E., Brunsteiner M., Fa M., Arancio O., Petukhov P., Thatcher G.R. (2013). Design, synthesis, and optimization of novel epoxide incorporating peptidomimetics as selective calpain inhibitors. J. Med. Chem..

[b0075] Schregel V., Jacobi S., Penin F., Tautz N. (2009). Hepatitis C virus NS2 is a protease stimulated by cofactor domains in NS3. Proc. Natl. Acad. Sci. U.S.A..

[b0080] Tedbury P.R., Harris M. (2007). Characterisation of the role of zinc in the hepatitis C virus NS2/3 auto-cleavage and NS3 protease activities. J. Mol. Biol..

[b0085] Thibeault D., Maurice R., Pilote L., Lamarre D., Pause A. (2001). *In vitro* characterization of a purified NS2/3 protease variant of hepatitis C virus. J. Biol. Chem..

[b0090] Welbourn S., Green R., Gamache I., Dandache S., Lohmann V., Bartenschlager R., Meerovitch K., Pause A. (2005). Hepatitis C virus NS2/3 processing is required for NS3 stability and viral RNA replication. J. Biol. Chem..

[b0095] Wyles D.L., Kaihara K.A., Korba B.E., Schooley R.T., Beadle J.R., Hostetler K.Y. (2009). The octadecyloxyethyl ester of (S)-9-[3-hydroxy-2-(phosphonomethoxy) propyl]adenine is a potent and selective inhibitor of hepatitis C virus replication in genotype 1A, 1B, and 2A replicons. Antimicrob. Agents Chemother..

