# Supplementary material for: Epoxide based inhibitors of the hepatitis C virus non-structural 2 autoprotease
Source: Antiviral Res. 2015 May;117:20–6. doi: 10.1016/j.antiviral.2015.02.005 (PMC4398321; doi:10.1016/j.antiviral.2015.02.005)
Supplement: Supplementary Fig. 1 — Activity against in vitro autoprotease activity. JFH1 NS2–3 refolding reactions were treated with a range of concentrations of 1–10 or cerulenin and NS3-FLAG proteolysis product (arrowhead) quantified by western blot. Determined EC50 values are shown in Table 1. [file mmc1.pptx]

## Slide 1
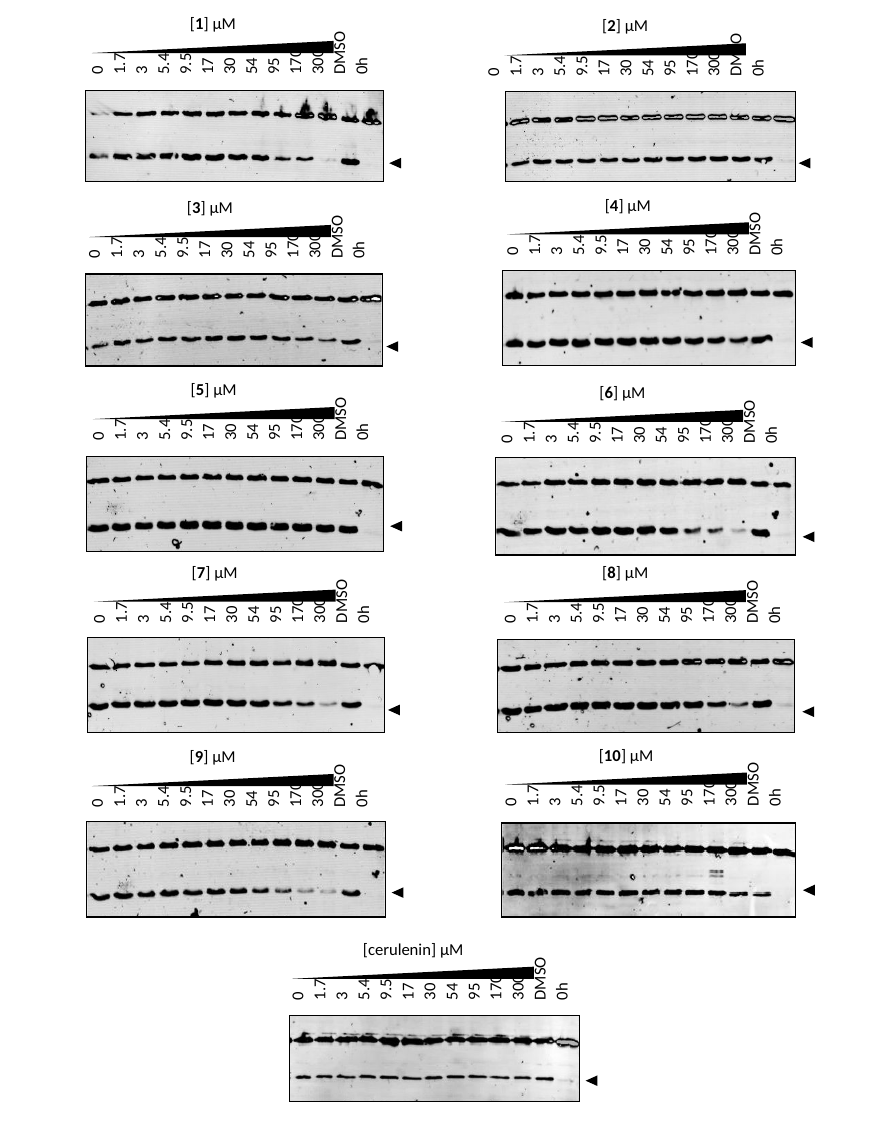

0
1.7
3
5.4
9.5
17
30
54
95
170
300
DMSO
0h
0
1.7
3
5.4
9.5
17
30
54
95
170
300
DMSO
0h
[1] µM
[2] µM
0
1.7
3
5.4
9.5
17
30
54
95
170
300
DMSO
0h
0
1.7
3
5.4
9.5
17
30
54
95
170
300
DMSO
0h
[4] µM
[3] µM
0
1.7
3
5.4
9.5
17
30
54
95
170
300
DMSO
0h
0
1.7
3
5.4
9.5
17
30
54
95
170
300
DMSO
0h
[5] µM
[6] µM
0
1.7
3
5.4
9.5
17
30
54
95
170
300
DMSO
0h
0
1.7
3
5.4
9.5
17
30
54
95
170
300
DMSO
0h
[7] µM
[8] µM
0
1.7
3
5.4
9.5
17
30
54
95
170
300
DMSO
0h
0
1.7
3
5.4
9.5
17
30
54
95
170
300
DMSO
0h
[10] µM
[9] µM
0
1.7
3
5.4
9.5
17
30
54
95
170
300
DMSO
0h
[cerulenin] µM
