# Supplementary figures and images for: Epoxide based inhibitors of the hepatitis C virus non-structural 2 autoprotease
Source: Antiviral Res. 2015 May;117:20–6. doi: 10.1016/j.antiviral.2015.02.005 (PMC4398321; doi:10.1016/j.antiviral.2015.02.005)

## Slide 1
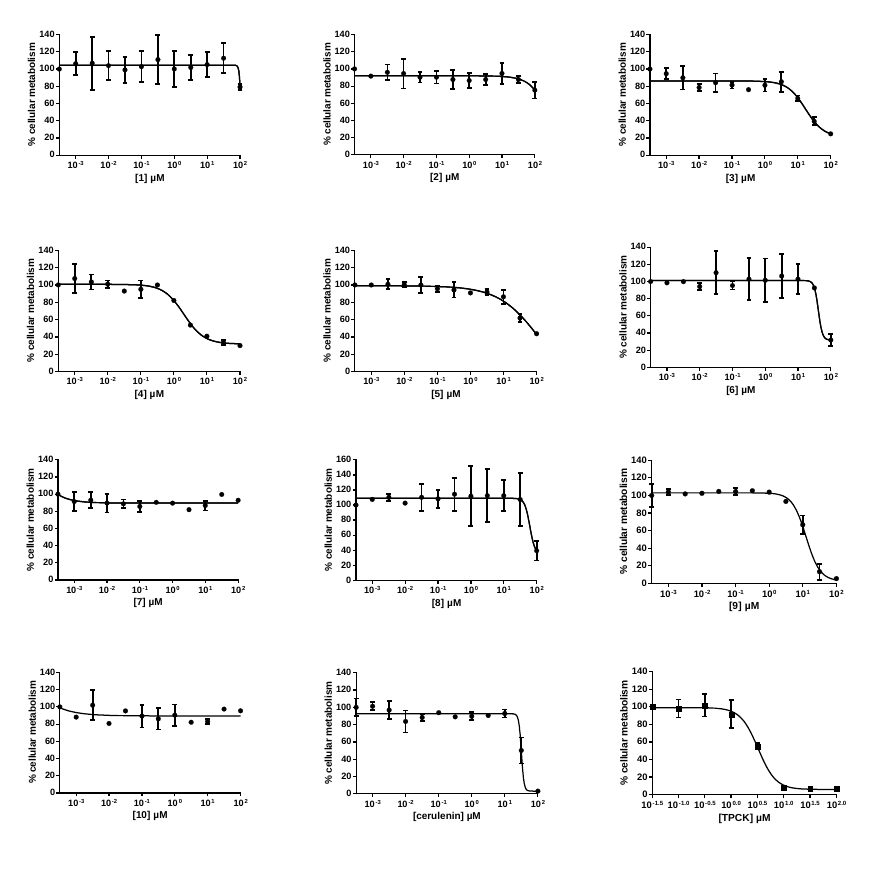

Supplement: Supplementary Fig. 2 — Cytotoxicity analysis of epoxide-based inhibitors of the NS2 autoprotease. Huh7 cells treated with indicated concentrations of 1–10, cerulenin or TPCK for 72 h (n = 2, average ± SD). Determined CC50 values for 1–10 and cerulenin are shown in Table 1. TPCK CC50 = 3.3 μM. [file mmc2.pptx]
