# Supplementary material for: Epoxide based inhibitors of the hepatitis C virus non-structural 2 autoprotease
Source: Antiviral Res. 2015 May;117:20–6. doi: 10.1016/j.antiviral.2015.02.005 (PMC4398321; doi:10.1016/j.antiviral.2015.02.005)
Supplement: Supplementary materials [file mmc3.docx]

**Supplementary Materials and Methods**

Recombinant NS2-3 expression and purification

NS2-3 was expressed in *E. Coli* BL21 (DE3) pLysS at 37 °C in LB medium containing 1 % (w/v) glucose and 50 µg/µl ampicillin, grown to OD_600_ = 1.0 before induction with 1 mM isopropyl-beta-D-thiogalactopyranoside (IPTG) for 3 h at 37 °C. Inclusion bodies were isolated in extraction buffer (6 M GdnHCl, 0.5 M NaCl, 50 mM Tris-HCl, pH 8.0) by centrifugation at 40,000 x g for 1 h. The resulting supernatant was loaded onto Chelating Sepharose^TM^ Fast Flow resin (GE Healthcare) charged with Ni^2+^ according to the manufacturer’s instructions. Loaded resin was washed with extraction buffer supplemented with increasing concentrations of imidazole, with NS2-3 eluted above 200 mM imidazole.

HCV sub-genomic replicon (SGR) stable cell lines

Constructs for HCV SGRs of the genotype 1b Con1 (BM4-5) and genotype 2a JFH1 isolates containing a firefly luciferase – neomycin phosphotransferase fusion protein (ffLuc - NPT) have been previously reported ([Wyles et al., 2009](#_ENREF_3), [Wyles et al., 2007](#_ENREF_4)), into which the relevant NS2 coding sequence was introduced. Constructs were linearised with XbaI, treated with mungbean nuclease to degrade 3’ overhangs and *in vitro* transcripts were produced using Ribomax Express (Promega) following the manufacturer’s instructions. Transcripts were purified by phenol/chloroform extraction and quantified by absorbance at 260 nm. 4 x 10^6^ Huh7.5 cells ([Blight et al., 2002](#_ENREF_1)) were electroporated with 10 µg RNA at 950 µF and 270 V. Polyclonal stable cell populations were selected by passage under 500 µg/ml G418 (Sigma Aldrich) and subsequently maintained in Dulbecco’s modified Eagle’s medium (DMEM) supplemented with 10 % FBS, 100 IU penicillin ml^-1^, 100 µg streptomycin ml^-1^, 1 % non-essential amino acids and 300 µg/ml G418 at 37°C in 5% CO_2_.

HCV SGR western blot analysis.

4 x 10^6^ SGR harbouring Huh7.5 cells or Huh7.5 cells containing JFH1 HCVcc were seeded in a 12-well plate (Corning) for 48 h before lysis in 500 µl Passive Lysis Buffer. Lysates were quantified using Pierce^TM^ BCA Protein Assay Kit (Thermo Scientific) following the manufacturer’s instructions. 30 µg protein was analysed by 15 % SDS-PAGE and Western blot using α JFH1 NS2 ([Jirasko et al., 2008](#_ENREF_2)) at 1:500, followed by IRDye 800CW Donkey anti-Rabbit secondary at 1:10,000 or αGAPDH (Abcam) at 1:20,000, followed by followed by IRDye 680RD Donkey anti-Mouse secondary (LI-COR Biosciences) at 1:10,000. Imaging was performed using Odyssey imager (LI-COR Biosciences).

BLIGHT, K. J., MCKEATING, J. A. & RICE, C. M. 2002. Highly permissive cell lines for subgenomic and genomic hepatitis C virus RNA replication. *J Virol,* 76**,** 13001-14.

JIRASKO, V., MONTSERRET, R., APPEL, N., JANVIER, A., EUSTACHI, L., BROHM, C., STEINMANN, E., PIETSCHMANN, T., PENIN, F. & BARTENSCHLAGER, R. 2008. Structural and functional characterization of nonstructural protein 2 for its role in hepatitis C virus assembly. *J Biol Chem,* 283**,** 28546-62.

WYLES, D. L., KAIHARA, K. A., KORBA, B. E., SCHOOLEY, R. T., BEADLE, J. R. & HOSTETLER, K. Y. 2009. The octadecyloxyethyl ester of (S)-9-[3-hydroxy-2-(phosphonomethoxy) propyl]adenine is a potent and selective inhibitor of hepatitis C virus replication in genotype 1A, 1B, and 2A replicons. *Antimicrob Agents Chemother,* 53**,** 2660-2.

WYLES, D. L., KAIHARA, K. A., VAIDA, F. & SCHOOLEY, R. T. 2007. Synergy of small molecular inhibitors of hepatitis C virus replication directed at multiple viral targets. *J Virol,* 81**,** 3005-8.
